# Antidiabetic Effects of Add-On *Gynostemma pentaphyllum* Extract Therapy with Sulfonylureas in Type 2 Diabetic Patients

**DOI:** 10.1155/2012/452313

**Published:** 2012-10-17

**Authors:** V. T. T. Huyen, D. V. Phan, P. Thang, P. T. Ky, N. K. Hoa, C. G. Ostenson

**Affiliations:** ^1^Endocrine and Diabetes Unit, Department of Molecular Medicine and Surgery, Karolinska Institute,17176 Stockholm, Sweden; ^2^Hanoi Medical University, 1000 Hanoi, Vietnam; ^3^National Institute of Gerontology, 1000 Hanoi, Vietnam; ^4^Department of Material Medica, Hanoi College of Pharmacy, 1000 Hanoi, Vietnam; ^5^Department of Internal Medicine, University of Manitoba, Winnipeg, Manitoba, Canada R3T 2N2

## Abstract

*Aims.* To investigate the antidiabetic effect of the traditional Vietnamese herb *Gynostemma pentaphyllum* (GP) together with sulfonylurea (SU) in 25 drug-naïve type 2 diabetic patients. *Methods.* After 4-week treatment with gliclazide (SU), 30 mg daily, all patients were randomly assigned into 2 groups to add on GP extract or placebo extract, 6 g daily, during eight weeks. *Results.* After 4-week SU treatment, fasting plasma glucose (FPG) and HbA_1C_ decreased significantly (*P* < 0.001). FPG was further reduced after add-on therapy with 2.9 ± 1.7 and 0.9 ± 0.6 mmol/L in the GP and placebo groups, respectively (*P* < 0.001). Therapy with GP extract also reduced 30- and 120-minute oral glucose tolerance test postload values. HbA_1C_ levels decreased approximately 2% units in the GP group compared to 0.7% unit in the placebo group (*P* < 0.001). *Conclusion.* GP extract in addition to SU offers an alternative to addition of other oral medication to treat type 2 diabetic patients.

## 1. Introduction

Diabetes mellitus is a metabolic disorder characterized by chronic hyperglycemia and type 2 diabetes (T2D) dominates with approximately 90% of the total diabetes population and with an increasing trend [1, 2]. Among oral hypoglycemic agents, sulfonylureas (SU) are still a cornerstone of treatment of T2D. In addition to metformin, SU is regarded as a particularly suitable first-line drug, especially when used relatively early following diagnosis [3]. However, along with duration of diabetes, patients treated with SU may develop secondary failure to the drug [4]. Monotherapies, including SU, are usually insufficient over long term in providing sustained normoglycemia for patients with T2D. For this reason, combination therapies are commonly required.

In Vietnam, traditional medicine, including herbs, has been rather popular for management of diabetes. *Gynostemma pentaphyllum* (GP) Makino (Family Cucurbitaceae) is a perennial creeping herb growing wild in the mountain regions of Vietnam, China and some other Asian countries. It has been used widely in Southeast Asian countries as an herbal medicine and being beneficial for the prevention and treatment of diabetes. However, these herbs have not been studied adequately. Our previous works on GP have revealed the anti-diabetic effect of GP extract in normal rats [5, 6]. Extract of GP has been shown to reduce both hyperglycemia and hyperlipidemia in diabetic Zucker Fatty rats [7]. A recent experimental study demonstrated that an ethanol extract of GP, produced in Vietnam, inhibited protein tyrosine phosphatase 1B (PTP-1B) activity, which may lead to enhanced insulin sensitivity and thereby improved glucose tolerance [8]. In addition GP extract has been proven in our recent clinical study to possess anti-diabetic effect with good safety data in newly diagnosed T2D patients, and to have effect on insulin sensitivity [9]. Thus, it is highly relevant to use GP extract as add-on therapy with SU in treatment of T2D.

The aim of the present study was to investigate the anti-diabetic effects and safety of add-on GP extract therapy with SU in a randomized, double-blind, placebo-controlled trial in drug-naïve type 2 diabetic patients. 

## 2. Research Designs and Methods

### 2.1. Medication

 Gliclazide was chosen as it is one of the most commonly prescribed SU. The add-on medication was provided in the form of GP extract at a dose of 6 g/day (3 g/packet, twice a day). Whole plants of *Gynostemma pentaphyllum* Makino-Cucurbitaceae were collected from the Hoa Binh province, in the north of Vietnam, and identified by Professor P. T. Ky, Department of Material Medica, Hanoi College of Pharmacy. The production of GP extract consisted two stages. The first stage was to authenticate the collected GP plants by comparison with the voucher specimen (HN-0152) deposited in the herbarium at the Department of Material Medica, Hanoi College of Pharmacy. The second stage was to produce the GP extract as specified. Briefly, the process included extraction of the authenticated GP plants for 2 h in boiling water, followed by the precipitation of impurities by addition of concentrated 70% ethanol. The 70% ethanol was then removed by distillation at low pressure, and impurities were removed by filtration. Thereafter, the extract was inspected and took the appearance of a semifinished brown powder with the typical odour of GP extract. This powder had a humidity of approximately 6.7%, and could be dissolved in water to produce a brown fluid with a sweet-bitter flavour. The extract contained, firstly, flavonoids, as shown by a positive cyanidin reaction with the base FeCl_3_ (5%) [10, 11], and secondly approximately 18% saponins, as indicated by a positive foaming test [10, 11]. Thus, the standardization of the GP extract preparations included a confirmation of typical odour, and sweet-bitter flavour, and the presence of approximately 7% humidity, positive reactions in the flavonoid (cyanidin) and saponin (foaming) tests. 

The placebo extract employed was green tea (*Camellia sinensis*), which was supplied at the same dose and was similar to the GP extract in shape and packaging. After grinding of the GP and placebo material the resulting soluble powder was packed in tin foil packets (6 × 5 cm) by an automatic packing machine. Each packet contained 3 g of powder, and 10 packets were packed in one box to be distributed to the patients. Both GP and placebo extract were easily dissolved in 60 mL water (room temperature) and taken 30 minutes prior to morning and evening meals.

### 2.2. Patients

The treatment options and possibility of beneficial effects and risks of the treatment were explained to newly diagnosed patients with T2D. Consecutive patients were enrolled into the study. Written informed consent was obtained from all subjects. The study protocol was approved by the research ethics board at Hanoi Medical University, Hanoi, Vietnam and the Regional Ethics Committee at the Karolinska Institutet, Stockholm, Sweden.

Inclusion criteria were: (1) newly diagnosed patients with T2D according to WHO criteria [12] were recruited at National Institute of Gerontology and two district hospitals in Hanoi, Vietnam; (2) age from 40 to 70 years old, (3) anti-diabetic drug naïve; (4) mean (of two) fasting plasma glucose (FPG) measurements from 9 to 14 mmol/L; (5) glycosylated hemoglobin (HbA_1C_) from 9 to 13%.

Exclusion criteria were: (1) previous pharmacological treatment for diabetes, (2) chronic complications related to type 2 diabetes, and (3) smoking subjects, (4) pregnancy or lactation, and (5) increased titres of GAD, and IA-2 antibodies.

Evaluation at baseline included detailed medical history and examination, fasting and oral glucose tolerance test,  HbA_1C_, liver and renal function tests, fasting lipid profile, insulin and C-peptide level. 

### 2.3. Study Designs

**Figure 1:**
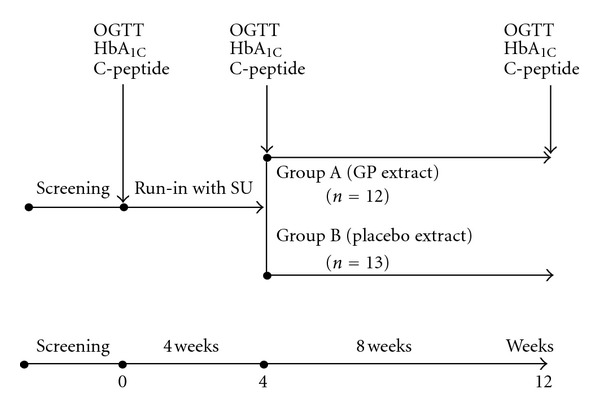
Study protocol. OGTTs (oral glucose tolerance tests), HbA_1C_, C-peptide, lipid, kidney, and liver function were performed at baseline, 4th, and 12th week. Fasting plasma glucose and insulin were determined every two weeks.

The protocol of this randomized, placebo-controlled, double blind clinical trial is presented in Figure 1. After screening, the selected subjects received gliclazide modified-release preparation 30 mg as single daily dose in 4 weeks. In addition, in 4-week run-in period, all patients were reinforced with lifestyle education, followed by concealed randomization. After four week-run in, all selected patients were randomly assigned by another, independent allocator by use of numbered containers into 2 groups, matched by age, sex and HbA_1C_: group A and group B received GP extract and placebo extract, respectively, in divided dose (twice a day, 30 minutes before breakfast and dinner). Group assignment of patients was blinded for both the main investigator and other investigators performing outcome analyses by use of a coding system, where the codes were kept by the independent allocator and revealed only after completing treatment periods and analyses. All subjects received comprehensive diabetes education, which included lifestyle and nutrition therapy. They were instructed to follow the diet as always recommended for newly diagnosed T2D patients and to walk 30 minutes a day and at least five days a week during the trial. This was reinforced at each follow-up visit, and subjects were treated on an outpatient basis. 

### 2.4. Primary and Secondary Outcomes

Primary outcomes are fasting plasma glucose and  HbA_1C_ (glycosylated hemoglobin), Homeostasis Model Assessment (HOMA) insulin resistance (HOMA-IR) and HOMA beta cell function (HOMA-*β*). Secondary outcomes are body mass index (BMI), weight, waist circumference, blood pressure, changes in blood lipids and liver enzymes, serum creatinine, and urea.

### 2.5. Biochemical and Anthropometric Analyses

Blood samples of fasting subjects were taken before, during (every second week for 12 weeks), and after the treatment course for measurement of plasma glucose, HbA_1C_, liver transferase (ALT, AST), urea, creatinine, lipid profiles, C-peptide, and insulin levels. Serum samples were obtained by centrifugation and stored at −20°C pending for assay. Analysis of glucose concentration of each sample was done by enzymatic colorimetric test, GOD-PAP in a glucose analyzer (Autolab Instrument, Boehringer Mannheim, Germany, wave-length Hg 546 nm). HbA_1C_ was measured with BIO-RAD D-10 (Bio-Rad Strasbourg, Schiltigheim, France). The insulin concentration was measured by insulin radioimmunoassay (RIA), using our own antibodies, human insulin as a standard, and charcoal addition to separate antibody-bound and free insulin [13]. C-petide was measured by human C-peptide RIA kit, HCP-20K, Millipore, 6 Research Park Drive, St. Charles, MO 63304, USA. Oral glucose tolerance tests (OGTT) (75 g glucose) were performed three times at baseline and after four and twelve weeks. Venous blood samples were drawn before (0), 30, and 120 minutes after the glucose intake. The Homeostasis Model Assessment (HOMA) was used to assess insulin sensitivity and beta cell function, based on fasting insulin and glucose levels and according to published algorithms: HOMA insulin resistance (HOMA-IR) = (insulin × glucose)/22.5, and HOMA beta cell function (HOMA-*β*) = 20 × insulin/(glucose − 3.5) [14]. Body weight, body mass index (BMI), waist and hip circumference, blood pressure, and registered adverse effects were noted in medical records during the visits. 

### 2.6. Statistical Analysis

Results are expressed as means ± SD. Paired *t*-test was used to analyze data in the same group before and after treatment with Bonferroni correction when multiple testing. The independent sample *t*-test was used for normal distributed variables to compare differences in mean change between the treatment group and the control group (SPSS version 16.0). Difference was considered significant if the *P* value was below 0.05.

## 3. Results

### 3.1. Clinical Characteristics

Twenty five subjects were enrolled in the study. The baseline characteristics of the patients are shown in Table 1. After 4-week treatment with SU, FPG significantly decreased from 11.4 ± 1.3 to 9.3 ± 1.3 mmol/L (*P* < 0.001). A similar decrement was observed with HbA_1C_ from 9.9 ± 1.0 to 8.9 ± 0.7% (*P* < 0.001). Increase in C-peptide and insulin levels, and improvements of lipid profile were also found (Table 1). 

At the fourth week, evaluations of all selected T2D patients revealed no statistically significant differences between groups allotted to treatment with GP and placebo extract regarding age, gender, systolic and diastolic blood pressure (SBP and DBP), body weight, BMI, waist, hip circumference, FPG, and HbA_1C_ (Table 2). All patients were compliant with the treatment protocol. 

### 3.2. Effects of Add-On GP Extract Therapy on Glucose Regulation

**Figure 2:**
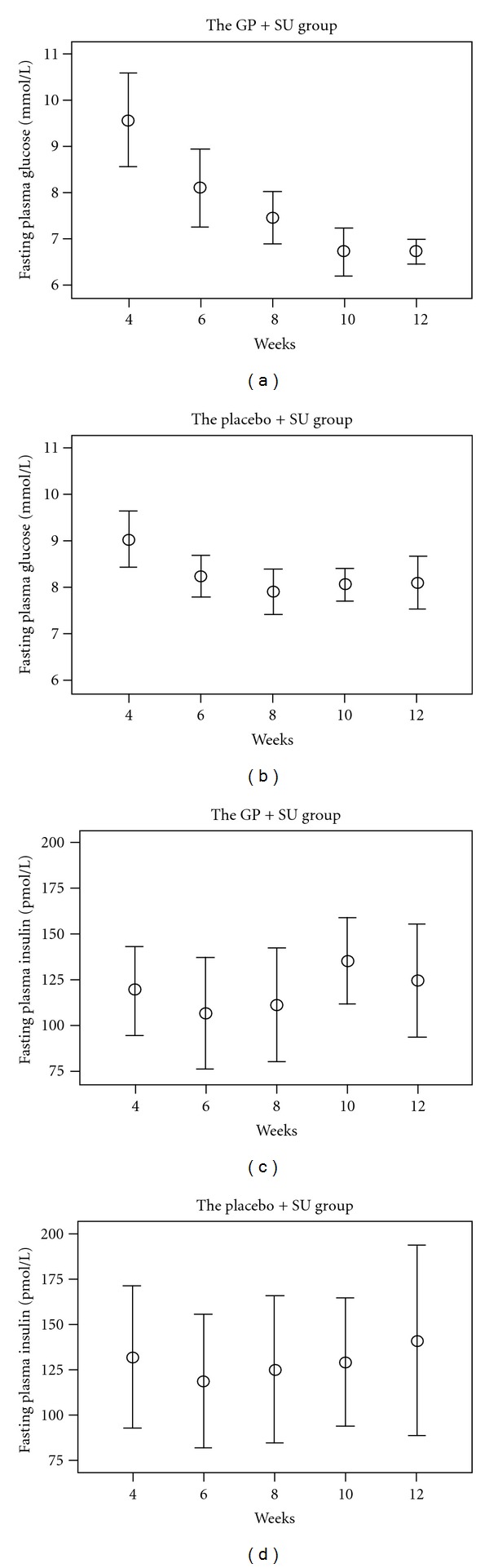
Fasting plasma glucose (mmol/L) and insulin (pmol/L) levels in GP (a) and (c) and placebo (b) and (d), resp.) groups after adding GP or placebo extract. Mean ± SD (*n* = 12 in GP group and *n* = 13 in placebo group).

In the GP extract group, FPG was not significantly different between groups after four weeks with add-on GP extract therapy (from 9.6 ± 1.6 to 7.5 ± 0.9 mmol/L, *P* = 0.188), but it was significantly decreased after six weeks of using add-on GP extract (*P* < 0.001) (Figure 2). At the end of the treatment course, the value of FPG totally decreased 2.9 ± 1.7 mmol/L to a level of 6.8 ± 0.4 mmol/L (*P* < 0.001). The levels of FPG in the placebo group were slightly reduced (0.9 ± 0.6 mmol/L, *P* < 0.001 versus reduction in GP group) at the end of treatment period to a level of 8.1 ± 1 mmol/L. Therapy with GP extract also significantly decreased 30 and 120 minute OGTT postload values (Table 3).

The HbA_1C_ values decreased from 9.0 ± 0.7 to 7.0 ± 0.7% in the GP group, and from 8.8 ± 0.6 to 8.1 ± 0.6% in the placebo group (*P* = 0.001). Thus, the decrement was significantly larger in the GP group than that in the placebo group (2.0 ± 0.9 versus 0.7 ± 0.5%-units, *P* = 0.001) (Table 3).

As shown in Table 3, the glycometabolic improvement was achieved without any major change of circulating insulin and C-peptide levels.

Change in HOMA-IR between fourth and twelfth week indicated that insulin resistance was not significantly decreased in the GP group (−2.3 ± 3.4) compared with that (−0.3 ± 5.8) in the placebo group (*P* < 0.311) and there were no significant changes in HOMA-*β* during eight weeks (Table 3).

### 3.3. Change in Body Weight and Other Parameters

There were no significant differences within or between groups regarding changes in serum triglyceride, total cholesterol, and HDL and LDL cholesterol levels. There were not significant changes on body weight from 54.9 ± 6.9 to 55.1 ± 6.5 kg in the GP group, and from 55.3 ± 11.4 to 54.8 ± 10.6 kg in the placebo group, during twelve weeks of treatment (Table 2). Similarly, no significant changes in the plasma levels of AST, ALT, creatinine, BUN, C-peptide, waist and hip circumferences, and blood pressure were detected during twelve weeks of treatment (Table 2). Adverse effects, such as gastrointestinal, diarrhea, and hypoglycemic symptoms, from the GP group and placebo group were not recorded during the research period and all of the patients completed the study.

## 4. Discussion

The results of the present study demonstrated improved glycemic control in patients treated with SU who received the addition of GP extract, as compared to patients on SU therapy alone. The decrease of HbA_1C_ was approximately 2%-units and of FPG nearly 3 mmol/L over 8 weeks. Thus, the glycemic control effect of the combination therapy with SU and GP extract was comparable with that of SU combined with metformin [15]. As the patients in both GP and placebo groups had similar diet and exercise regimes, the GP extract, but not diet and exercise, was mainly responsible for the additional improvement of glycemic control. Green tea (*Camellia sinensis*) was reported to induce hypoglycemic effect in streptozotocin-diabetic rats [16] but there has been little evidence in improving human glycemic control [17, 18].

The improvement of diabetic control with the combined therapy occurred without any significant change of insulin and C-peptide levels after adding GP extract to the stimulating insulin SU, supporting that GP extract may act preferentially by improving insulin sensitivity, as was indicated by analysis of HOMA-IR [9]. In the present study, HOMA-IR tended to decrease more than in the placebo group; however the difference was not significant. With respect to these observations, it appears that GP extract may provide improved glycemic control via a mechanism that is independent of stimulation of insulin release and that does not place any additional burden on defective *β*-cells. An effect by GP extract on insulin sensitivity may be accounted for by suppression of PTP-1B activity present in liver and skeletal muscle by dammarene compounds in the GP extract [8]. Given the possibility that GP extract might have effect on insulin sensitivity, more understanding is needed about the appropriate combination of GP extract and other anti-diabetic medication as well as about the differences in the potential risks and benefits to people with T2D. From this knowledge, ways to maximize benefit and minimize risk can be ascertained.

SU plus GP extract combination was generally well-tolerated. No adverse effects were documented in the combination treatment with SU and GP extract compared with SU as monotherapy or SU and placebo extract. Compared with the most common combination SU plus metformin, no patients experienced adverse gastrointestinal effects including diarrhea, nausea, epigastric discomfort, and anorexia, which might be the causes of treatment discontinuation in some studies [19, 20]. In addition, we did not see any weight gain in both groups during twelve weeks of treatment, therapy with GP extract might be weight neutral in patients with T2D and limit the weight gain experienced with SU. This weight neutral effect can be observed in the combined therapy between metformin and SU [21]. In the study, neither hepatoxicity nor nephrotoxicity developed, in support of the biosafety of GP extract used as add-on therapy with SU. As a result, indeed, the study had an ideal compliance (100%). 

We used a low dose of the SU, gliclazide, and no patient experienced symptoms of hypoglycemia even when used in the combination therapy with GP extract. The risk of hypoglycemia often increases in parallel with dose increment of SU [22], however, with the addition of GP extract, the observed results proved that this extract can be efficacious for the maintenance of the effects of low dose of SU treatment.

## 5. Conclusion

In conclusion, as add-on therapy to SU, GP extract improved glycemic control and this improvement was sustained over 12 weeks. Furthermore, there were potential benefits in terms of maintaining low-dose SU without symptoms of hypoglycemia. The biosafety of extract was suggested clinically, since no acute hepatoxic and nephrotoxic or other adverse effects were observed in the trial. Therefore, our results could offer an alternative to addition of other oral medication to treat type 2 diabetic patients using traditional Vietnamese medicine.

## Figures and Tables

**Table 1:** Baseline clinical characteristics and laboratory findings of the patients.

	Baseline	After 4 weeks
*n*	25	25
Age (years)	55.0 ± 9.0	55.0 ± 9.0
Sex (Male : Female)	15 : 10	15 : 10
Body weight (kg)	55.0 ± 9.6	55.1 ± 9.3
BMI (kg/m^2^)	22.1 ± 3.6	22.2 ± 3.5
Waist (cm)	86.6 ± 8.9	86.6 ± 9.1
Hip (cm)	96.9 ± 6.6	96.4 ± 6.8
Systolic blood pressure (mmHg)	120.8 ± 7.6	120.4 ± 9.5
Diastolic blood pressure (mmHg)	78.6 ± 4.9	77.8 ± 5.4
Fasting plasma glucose (FPG) (mmol/L)	11.4 ± 1.3	9.3 ± 1.3*
Fasting plasma insulin (pmol/L)	99.0 ± 33.0	126.0 ± 55.8**
30-minute plasma insulin (pmol/L)	190.8 ± 113.4	267.6 ± 136.8**
120-minute plasma insulin (pmol/L)	303.0 ± 244.2	457.2 ± 233.4**
HbA_1C_ (%)	9.9 ± 1.0	8.9 ± 0.7*
C-peptide (ng/mL)	0.8 ± 0.6	1.6 ± 0.8*
30-minute C-peptide (ng/mL)	1.4 ± 1.2	2.6 ± 1.3*
Cholesterol (mmol/L)	5.1 ± 1.2	4.5 ± 0.9**
Triglyceride (mmol/L)	3.0 ± 2.2	2.2 ± 1.3**
HDL-cholesterol (mmol/L)	1.2 ± 0.2	1.2 ± 0.3
LDL-cholesterol (mmol/L)	2.9 ± 0.8	2.5 ± 0.8**

Results are means ± SD, *P < 0.001, **P < 0.01.

**Table 2:** Clinical characteristics and laboratory findings of the patients at week 4 and 12.

	GP group		Placebo group	
	Week 4	Week 12	Week 4	Week 12
*n*	12	12	13	13
Age (years)	55.6 ± 9.9	55.6 ± 9.9	54.5 ± 8.4	54.5 ± 8.4
Sex (Male : Female)	7 : 5	7 : 5	8 : 5	8 : 5
Body weight (kg)	54.9 ± 6.9	55.1 ± 6.5	55.3 ± 11.4	54.8 ± 10.6
BMI (kg/m^2^)	21.8 ± 2.7	21.9 ± 2.7	22.5 ± 4.2	22.3 ± 4.0
Waist (cm)	85.8 ± 5.9	85.3 ± 5.6	87.3 ± 11.5	86.8 ± 10.7
Hip (cm)	95.3 ± 4.3	95.1 ± 4.2	97.3 ± 8.5	96.4 ± 8.4
Systolic blood pressure (mmHg)	123.5 ± 9.1	118.3 ± 9.4	119.2 ± 6.4	120.8 ± 9.5
Diastolic bood pressure (mmHg)	79.6 ± 5.3	75.4 ± 7.2	76.2 ± 5.1	77.7 ± 6.0
Fasting plasma glucose (FPG) (mmol/L)	9.6 ± 1.6	6.7 ± 0.4*	9.0 ± 1.0	8.1 ± 1.0
Fasting plasma insulin (pmol/L)	120.0 ± 42.0	124.6 ± 49.0	131.4 ± 67.2	139.0 ± 89.9
HOMA-IR	8.5 ± 3.0	6.2 ± 2.4	9.0 ± 5.2	8.7 ± 6.4
HOMA-*β*	69.2 ± 28.4	131.5 ± 55.4	78.4 ± 38.4	97.3 ± 54.3
HbA_1C_ (%)	9.0 ± 0.7	7.0 ± 0.7*	8.8 ± 0.6	8.1 ± 0.6
C-peptide (ng/mL)	1.7 ± 0.7	1.8 ± 0.8	1.5 ± 0.8	1.8 ± 1.2
30-minute C-peptide (ng/mL)	2.2 ± 1.3	2.7 ± 1.3	2.9 ± 1.3	3.2 ± 2.0
Cholesterol (mmol/L)	4.8 ± 1.1	4.9 ± 1.0	4.3 ± 0.8	4.3 ± 0.9
Triglyceride (mmol/L)	2.4 ± 1.5	2.3 ± 1.1	2.0 ± 1.2	2.0 ± 1.4
HDL-cholesterol (mmol/L)	1.3 ± 0.4	1.2 ± 0.2	1.1 ± 0.2	1.1 ± 0.3
LDL-cholesterol (mmol/L)	2.8 ± 0.9	2.9 ± 0.6	2.3 ± 0.6	2.3 ± 0.6

Results are means ± SD, *P < 0.001.

**Table 3:** Changes in plasma glucose, HbA_1C_, HOMA-IR, HOMA-*β*, insulin, and C-peptide levels from week 4 to 12.

	GP group *n* = 12	Placebo group *n* = 13	*P*
Change in FPG (mmol/L)	−2.9 ± 1.7	−0.9 ± 0.6	<0.001
Change in 30 minutes plasma glucose (mmol/L)	−1.8 ± 3.0	+1.2 ± 2.7	0.015
Change in 120 minutes plasma glucose (mmol/L)	−3.6 ± 6.3	+1.3 ± 2.8	0.017
Change in fasting plasma insulin (pmol/L)	4.3 ± 56.7	7.8 ± 90.9	0.911
Change in 30 minutes plasma insulin (pmol/L)	75.4 ± 139.2	5.4 ± 119.4	0.193
Change in 120 minutes plasma insulin (pmol/L)	7.2 ± 270.6	−68.4 ± 170.4	0.411
Change in fasting plasma C-peptide (ng/mL)	0.12 ± 0.91	0.29 ± 1.46	0.714
Change in 30 minutes C-peptide (ng/mL)	0.52 ± 1.79	0.29 ± 1.93	0.774
Changes in HbA_1C_	−2.0 ± 0.9	−0.7 ± 0.5	<0.001
Change in HOMA-IR	−2.3 ± 3.4	−0.3 ± 5.8	0.311
Change in HOMA-*β*	62.3 ± 59.3	18.8 ± 61.2	0.085

Results are means ± SD of patients in each group.
